# High Incidence of Workplace Violence in Metropolitan Emergency Departments of Thailand; a Cross Sectional Study

**DOI:** 10.22037/aaem.v9i1.1140

**Published:** 2021-03-25

**Authors:** Adisak Nithimathachoke, Wanawat Wichiennopparat

**Affiliations:** 1 Department of Emergency Medicine, Faculty of Medicine Vajira Hospital, Navamindradhiraj University, Bangkok, Thailand 10300.

**Keywords:** Emergency department, Factors, Hospital, Personnel, prevention measure, violence

## Abstract

**Introduction:**

Violence against healthcare workers mostly occurs in emergency departments and is a serious global public health issue. This study aimed to evaluate the prevalence of violence directed towards emergency department healthcare personnel and to ascertain the factors that might be correlated with it.

**Methods:**

In this cross-sectional study, an anonymous questionnaire was used to gather data from healthcare personnel working in the emergency departments under the direction of the Bangkok Metropolitan Administration between 1 August 2019 and 30 November 2019, regarding the experience of violence during the previous year.

**Results:**

A total of 258 (87.5%) responses were received from 295 personnel. The results showed that 88.4% (228 personnel) had experienced violence during the past year, of these, 37.6% involved physical abuse that caused minor injuries. Employees with shorter tenures, nurses, and those working in tertiary academic emergency departments in the central business district were found to have increased likelihood of confronting violence. Measures taken to prevent violence had a limited impact on the occurrence rate. The most common impact on employees after experiencing violence was discouragement in their jobs (75.1 %). The key factors that promoted cases of violence were the consumption of alcohol or drugs (81.3%) and long waiting times (73.6%). Most violence tended to occur during non-office hours (95.4%). One-third of emergency healthcare personnel reported facing violence during their work.

**Conclusions:**

Emergency healthcare personnel in metropolitan of Thailand had a high rate of experiencing violence in the previous year. Younger age, lower work experience, being a nurse, and working in the urban academic or tertiary emergency department increased the likelihood of being a victim of workplace violence.

## Introduction

Healthcare providers are more likely to experience workplace violence than any other service occupations. Additionally, workplace violence in the health sector is a global public health issue (1, 2). Emergency departments (ED) are recognized as a high-risk area for violence against healthcare personnel and many studies reported a high occurrence of aggression initiated by patients or their relatives (3-7). An emergency department has many factors that might aggravate violent behaviors: long waiting times, crowding, various patient conditions, and substance use (8, 9). Workplace violence is related to job dissatisfaction, burnout, and turnover rate (10). Violence also results in psychiatric problems and physical injury, which impact both the personnel and their organizations and ultimately affect the care of patients.

Many works of literature emphasize using tools to minimize violence such as risk assessment, incident reports, and security systems (5, 11-15). Most of them show inconclusive results in practice, though statistically significant outcomes in training sections have been reported in some studies (16-20). Laws have been passed to help mitigate the issue in some regions. However, the incidence of violence directed toward healthcare personnel continues to increase (21-24). 

A study on violence against nurses working in emergency department was published 10 years ago, and only one rural and one urban emergency department were included (6). Our study aimed to examine the occurrence rates and characteristics of violence directed at healthcare professions in the EDs of metropolitan Thailand. Moreover, the use of preventive measures and factors inducing violence were also explored. 

## Methods

**Study design and setting**

In this cross-sectional study, an anonymous questionnaire was used to gather data from healthcare personnel working in the emergency departments under the direction of the Bangkok Metropolitan Administration, regarding the experience of violence during the previous year. The data was collected between 1 August 2019 and 30 November 2019, after receiving approval from Vajira Institutional Review Board (VIRB), Faculty of Medicine, Vajira Hospital, Navamindradhiraj University and Bangkok Metropolitan Administration Human Research Ethics Committee (BMAHREC) (COA 036/2561) on November 7, 2018. Once permission from the directors of participant hospitals was acquired, the study was explained to heads of each emergency department and all eligible participants. Permission requests from individual participants were made simultaneously.

**Participants**

According to the report from the Health System Research Institute of Thailand, the study population consisted of 3,000 healthcare personnel working in emergency departements of goverment hospitals in Bangkok,Thailand. In the previous study, 84.7% of healthcare personnel suffered from violence in the emergency department (6). Therefore, the minimum number of respondents in this survey was determined at 184 with a 95% confidence interval and 5% margin of safety. Considering the potential of missing data or non-respondents, an additional 10% was added to the number of participants required to be enrolled. The total amount of The total amount of respondents who were needed came to 203 healthcare personnel came to 203 healthcare personnel. We selected all 9 EDs of hospitals under the direction of the Medical Service Department of Bangkok Metropolitan Administration as the targets for the survey, which was conducted as a traditional paper-based survey. 

Healthcare providers who worked in emergency departments at least 40 hours a week with at least 1-year of work experience in the emergency departments were eligible to participate. However, the full-time personnel who were not providing care for patients were considered to be excluded from the study. Following the criteria, a total of 295 providers were qualified to take part in the survey, from whom we intended to collect data without randomization.

**Definition of violence**

Violence in this study was comprised physical assault and psychological assault. A physical assault was the use of physical force with or without an object against a person to threaten or harm them i.e. punch, kick, bite, and push. A psychological assault, without the use of physical force, was defined as an act against another person’s mental well-being and included verbal threat, harassment, criticism, etc. 

The workplace violence definitions in this study were assimilated with the definitions outlined by the World Health organization, which consisted of physical violence and verbal violence. Physical violence was the use of physical force with or without an object against a person to threaten or harm i.e. punch, kick, bite, and push. Verbal violence referred to the use of comments that were known to be humiliating, embarrassing, offensive, threatening, or degrading to another person including swearing and insults.

**Data collection**

Questionnaires with instructions alongside contact information of the researchers were sent to the heads of the emergency departments. All responses in this survey were anonymous, the questionnaires were treated as confidential and it was impossible to trace back any data.

An anonymous self-administrated questionnaire was modified from Workplace violence in health sector country case studies research instruments survey questionnaire by ILO/ICN/WHO/PSI Geneva 2003 (25) and reviewed by two members of faculty specializing in emergency medicine who were not involved in the study. Testing was conducted with 10 providers who had experience in the emergency department to check whether the questionnaire was clear and could be understood correctly. Subsequently, revisions were made for clarity. The questionnaire comprised 4 parts: demographic data, characteristics of violent incidents, consequences, and prevention measures in the emergency department.

**Data analysis**

The data was analyzed using IBM Corp. Released 2013. IBM SPSS Statistics for Windows, Version 22.0. Armonk, NY: IBM Corp. The quantitative data were reported as mean and standard deviation, t-test was used in normal distribution and Mann-Whitney U test in non-normal distribution to analyze the correlation. The categorical data such as gender, working experience, and type of violence were reported in numbers and percentage. The chi-square test or Fisher’s exact test was implemented to examine the correlation between each factor and experiences of violence where p-value < 0.05 represented statistical significance. Logistic regression was performed to analyze the odds ratio for the statistically significant independent variables.

## Results

**Baseline characteristics of participants**

The total response rate was 87.5% (258 out of 295 questionnaires). The participants were 60 physicians (23.3%), 187 nurses (72.5%) and 11 nurse aids (4.3%). The mean age of all participants was 31.2 years (S.D. = 8.16) and 78.3% were female. Most of the participants were under 30 years of age with average work experience of 7.4 years (S.D. = 7.24). The most common amount of working experience among participants was 1 year. Tertiary hospitals and one university hospital located in the central district area of Bangkok were the workplaces of 68.2% of participants. The rest of the participants were working in secondary care hospitals located in the periphery of Bangkok. Although 87.5% completed the questionnaires, there were 37 non-respondents (including the incomplete questionnaires) in this survey, more than half of whom (57.8%) were working in the secondary hospitals.

**Workplace violence**

Table 1 summarizes the characteristics, consequences, and aggravating factors of violence among studied participants. 88.4% of medical personnel were assaulted during the previous year. Psychological violence happened far more than physical violence. Even though psychological assistance was not considered necessary by any of the respondents, nearly half of them expressed feelings of discouragement to work in an emergency department. Physical impacts were minor injuries for which medical treatment was not necessary. Most of the violence occurred during non-office hours, while only 4.6% of the violence occurred in the morning shift (8 a.m. to 4 p.m.). The respondents stated that the triage area and treatment area were the places where most violence occurred. More than half of the participants claimed that drunkenness, long waiting times, crowding, and disease-related factors were contributing to violence. The answers to the open-ended question about violence aggravating factors were negligence of the administrative persons, vague laws on this issue, and social media effects. Less than half of the medical personnel (36.0%) used their hospitals’ incident reporting systems, which were not useful in respondents’ perspectives.

Workplace violence, composed of physical assaults and verbal abuse, had occurred in case of the majority of participants during the previous year. Among the three professions, the ratios of being subjected to physical violence were lowest in physicians (20%). Whereas, 72.7% of nurse-aides were injured from both forms of violence. Physical violence happened more in younger participants and who had less work experience. The variables that had a statistically significant association with the experience of both types of violence were age, job tenure, profession, and type of hospital. In contrast, the incidence rate of both categories of violence was not different between males and females (Table 2).

**Correlations**

The relationship between age (p = 0.007), profession (0.031), work experience (p = 0.014), and type of emergency department (p = 0.026) and frequency of workplace verbal and physical violence was found to be statistically significant. The younger emergency providers and individuals who had less work experience tended to face workplace violence significantly more compared to the providers between 51-60 years of age and those who had worked in the emergency department for more than 10 years. 

Nurses were prone to experience workplace violence more than other professions (OR: 6.143; 95% CI: 1.460 to 18.078). The emergency personnel who worked in the tertiary (OR: 2.746; 95% CI: 1.115 to 6.823) and in the university emergency departments (OR 2.431; 95% CI: 1.059 to 7.769), located in the central business district of Bangkok, were more likely to experience violent acts than were those working in secondary emergency departments, which were located in the periphery of Bangkok (Table 3).

**Prevention measures**

The prevention measure presented in most EDs was the authorized access entrance (81.4%). However, less than half of the participants (32.6%) reported having security guards at the entrance of their EDs and 8.5% had police activating systems. None of the respondents reported having any weapon screening measures before patients or visitors entering the EDs. Nonetheless, there was no association between having security systems and experience of physical violence. Furthermore, all of the participants lacked training in workplace violence and there were no protocols for prevention or mitigation of aggression in their organizations (Table 4).

## Discussion

Our study results show a significantly high rate of being violated amongst healthcare personnel working in metropolitan EDs in Thailand. This is consistent with the results from multinational studies (4, 8, 9, 19, 24-27). Though workplace violence in the health sector is 15% to 20% higher than the other industries (2, 27), only 8-38% of providers in other healthcare settings experienced workplace violence (1, 28). The study in 2008 in Southern Thailand with 545 participants also found that only 38.9% of the nurses working in all departments were abused by verbal violence while 3.1% of them suffered from physical violence (29). The figures from the mentioned studies imply that an emergency department is a place where most violence in the hospital occurs. 

Though none of the respondents had any serious physical injury from any weapon, the aggressions from patients or relatives are not acceptable. Ignoring minor violence and verbal abuse could foster an environment that encourages more serious criminal events as stated in the broken windows theory (30). Furthermore, the empirical results of this study suggested that when faced with violent acts, the participants mostly felt discouraged to continue working in the emergency departments. As stated in previous studies, these emotional consequences caused by violent experience could lead to depression, burnout syndrome, and eventually, drive the personnel to quit their jobs (10, 19, 31). Moreover, a healthcare worker with emotional distress is more likely to be a victim of violence (27). 

The minority of the participants reported every time they had been violated, showing that the perception of the usefulness of the reporting system in Thailand has not changed for years (6, 29). According to Stene J.’s study, emergency department personnel perceived violence as part of their job, dismissing the opportunities to improve the risk reduction system. Therefore, it is necessary to educate healthcare service providers about risk prediction, risk management, and risk report system as well as when to take legal action (5). 

Non-office hours, specifically between 4 p.m. to midnight, were the time that almost all the violence occurred. According to the study by Ferri P. et al., the evening shifts face inadequate manpower problems; and compared with other periods, more drunk and confused patients come to emergency departments during this period (32). These characteristics of emergency departments in non-office hours were similar to the violence aggravating factors specified by the respondents in the present study. In the previous studies in Thailand, the majority of violent acts had happened during non-office hours. Moreover, the factors that triggered most violence were similar to this study (1, 6, 29, 32). The report from the American College of Emergency Physicians (ACEP) (13) suggested that factors that promoted violence tended to increase upon the growth of delinquency and drug consumption. Other contributing factors included healthcare facilities’ inadequacy in providing psychiatric counseling services during non-office hours, inability to admit psychiatric patients as an inpatient, and refusal to grant patient’s request for specific treatment and medication. Dynamic management could be more helpful than fixed security measures such as increasing manpower during high patient volume periods, reducing waiting times, setting up a protocol for dealing with drunkenness, etc.

In line with previous studies in the emergency departments, age, and work experience affected the likelihood of being a victim of violence among the emergency personnel, but males and females were similarly being subjected to workplace violence (3, 8, 26, 27). This finding was contrary to those of Kowalenko T.’s research on violence against medical service providers in America and a study in India, which stated that female healthcare personnel were more prone to experiencing physical violence than their male counterparts (33, 34). In the present study, nurses were most likely to be faced with workplace violence, which is consistent with previous reports in Egypt and suburban EDs in Thailand (8, 35). The emergency providers who were younger and individuals who had less work experience confronted significantly more workplace violence compared to the more experienced group and those aged between 51-60 years, the reason for which could be the difference in total work hours each week and the pattern of shift work (35).

Though all of the emergency departments in this study were in the metropolitan of Thailand, we found that working in tertiary and university emergency departments located in the central business district area increased the likelihood of being violated. This might be the effect of the patient volume. More than 50,000 visits annually at each tertiary emergency department and around 40,000 visits annually at each secondary facility. Nevertheless, the results could have been different considering the number of non-respondents, more than half of whom worked in secondary hospitals. Providers in our study encountered far more violence compared with 61.7\% of violence in 472 participants in Thai suburban emergency departments (35). Currently, the data are inconclusive regarding location and type of emergency departments as risk factors. The emergency physician working in the high volume academic EDs or the state EDs in Turkey are more likely to be a victim of workplace violence (27). Whereas, 86\% of Australian nurses in the rural hospitals experienced violence compared to 43\% of nurses working in the urban hospitals (36). Besides, the healthcare providers in secondary level hospitals in China are more susceptible to aggression than those in primary and tertiary hospitals (37). Nevertheless, a prospective study found no association between the level of hospital and experience of violence (38). 

The majority of emergency physicians in Bangkok experienced violence, most of which were verbal abuse. Merely, 20% of these physicians encountered physical violence while 7.7% of physicians who were working in EDs in the suburban area of Thailand had faced physical violence (35). This number is relatively low compared with 38.4% of emergency physicians in the United States who had experienced more physical violence based on a survey in 2018 (39). Additionally, data from national judgment documents of China showed that doctors were the target group of violence and the majority of them were physically abused (4). These differences in results might be due to the different social structures such as the number of delinquencies, consumption of drugs, the law allowing citizens to carry weapons. 

Although guidelines recommend using security tools to minimize workplace violence in the emergency departments (13, 14), not all of the emergency departments had these tools. Particularly, none of the emergency departments in this study had a metal detector or weapon screening. However, none of the participants had experienced violence using any weapons. This is similar to prior researches in EDs of Thailand and Italy (6, 35, 40). Moreover, we found no relationship between having security systems and being a victim of aggression. While metal detectors markedly increased the rate of weapon detection, its impact on the occurrence of violence was not well established (19, 27). Also, the presence of security guards does not decrease the incidence of violence (16). Prediction of the aggressors and de-escalation methods have been used in hope of violent act prevention; however, no explicit data supports the efficacy of these measures (20). The complexity of workplace violence in emergency departments is well recognized. The actual occurrence rate and event details are important so an incident report should be emphasized. Also, a comprehensive hazard analysis with multifaceted measures should be used in conjunction with support from executive authorities. The aggravating factors should be corrected simultaneously with the use of other prevention methods. Additionally, quality improvement measures should be used to evaluate and improve the results.

**Table 1:** Characteristics, consequences, and aggravating factors of violence among studied participants

**Variable **	**Number (%)**
**Victim of violence**	
Psychological violence	218 (85.7)
Physical violence	93 (37.6)
**Emotional consequence**	
Anger	133 (51.6)
Desire to quit the job	107 (41.4)
Wish to work outside ED	87 (33.7)
Sadness	78 (30.2)
Shame	65 (25.2)
**Physical consequence**	
Abrasion	64 (24.8)
Contusion	35 (13.5)
**Work shift when violence occurred**	
Morning shift (8 am. To 4 pm.)	12 (4.6)
Evening shift (4 pm. To midnight.)	189 (73.3)
Night shift (midnight. To 8 am.)	57 (22.1)
**Contributing factors**	
Drunkenness or drug consumption	210 (81.3)
Long waiting time	190 (73.6)
Crowding	167 (64.7)
Symptom or disease	130 (50.4)
Inadequate security system	100 (38.8)
Miscommunication	98 (38.0)
Stressful situation	79 (30.6)
Unexpected treatment result	67 (26.0)
Improper waiting area	50 (19.4)
Lack of privacy	35 (13.6)
**Area the violence occurred**	
Triage area	135 (52.3)
Treatment area	107 (41.5)
Waiting area	16 (6.2)
**Every violence was reported**	
Yes	82 (36.0)
No	165 (64.0)

*More than one answer per question was acceptable. ED: emergency department.

**Table 2:** Correlation between baseline charactericts of participats and frequency of experiencing different types of violence

**Variable**	**Type of violence**					
	Both* (n=228)	**P**	**Verbal (n=218)**	**P**	**Physical (n=93)**	**P**
**Age (year)**						
21-30	144 (92.3)	0.007	136 (87.2)	0.047	68 (43.6)	0.016
31-40	59 (88.1)		58 (86.6)		18 (26.9)	
41-50	21 (72.4)		20 (69)		6 (20.7)	
51-60	4 (66.7)		4 (66.7)		1 (16.7)	
**Gender **						
Female	179 (88.6)	0.818	170 (84.2)	0.838	72 (35.6)	0.875
**Profession **						
Physician	48 (80.0)	0.031	43 (71.1)	0.005	12 (20.0)	0.001
Nurse	171 (91.4)		167 (89.3)		73 (39.0)	
Nurse aide	9 (81.8)		8 (72.7)		8 (72.7)	
**Experience (year)**						
1-5	93 (91.2)	0.014	86 (84.3)	0.021	45 (44.1)	0.038
5-10	90 (91.8)		89 (90.8)		34 (34.7)	
> 10 years	45 (77.6)		43 (74.1)		14 (24.1)	
**Type of hospital **						
University	71 (92.2)	0.026	70 (90.9)	0.004	27 (35.1)	0.003
Tertiary	91 (91.9)		88 (88.9)		47 (47.5)	
Secondary	66 (80.5)		60 (73.2)		19 (23.2)	

*: verbal + physical. Data are presented as number (%).

**Table 3:** Predictors and the likelihood of experiencing workplace violence during the previous year among studied cases

**Variable**	**Experience of violence**		**P**	**OR**	**95% CI**
	**No (n=30)**	**Yes (n=228)**			
**Age (year)**					
21-30	12 (4.7)	144 (55.8)	0.007	6.011	0.995 - 36.176
31-40	8 (3.1)	59 (22.9)		3.687	0.579 - 23.476
41-50	8 (3.1)	21 (8.1)		1.312	0.220 - 8.624
51-60	2 (0.8)	4 (1.6)		Ref	
**Profession **					
Physician	12 (3.1)	48 (20.2)	0.031	1.809	1.024-11.437
Nurse	16 (7.8)	171 (64.7)		6.143	1.460-18.078
Nurse aide	2 (0.8)	9 (3.5)		Ref	
**Work experience (year)**					
1-5	9 (3.5)	93 (36.1)	0.014	2.848	1.102-7.360
5-10	8 (3.1)	90 (34.9)		3.010	1.145-7.917
> 10	13 (5.0)	45 (17.4)		Ref	
**Type of Hospital **					
University hospital	6 (2.3)	71 (27.5)	0.026	2.431	1.059-7.769
Tertiary hospital	8 (3.1)	91 (35.3)		2.746	1.115-6.823
Secondary hospital	16 (6.2)	66 (25.6)		Ref	

OR: Odds Ratio, Ref: reference, CI: connfidence interval. Data are presented as number (%).

**Table 4:** Relationship between existence of preventive measures and experience of physical violence

**Violence prevention system**	**N (%)**	**Experience n (%)**		**P**
		**Yes **	**No**	
**Authorized access entrance**				
Yes	210 (81.4)	79 (30.6)	131 (50.8)	0.319
**Guard at ED entrance**				
Yes	84 (32.6)	27 (10.5)	57 (22.1)	0.364
**Guard inside ED**				
Yes	14 (5.4)	9 (3.5)	5 (1.9)	0.979
**Police activating system**				
Yes	22 (8.5)	9 (3.5)	13 (5.0)	0.619

ED: emergency deparment; data are presented as number (%).

## Limitations

Potential recall bias is the main limitation of this study. The questionnaires were developed and tested based on theory but it must be taken into account that the participants might answer each question based on their own interpretation. The participants were just a part of emergency department healthcare workers in Bangkok, Thailand and the actual occurrence rate might be different. Besides, the individuals’ number of working hours and shift work patterns could affect their experience of violence, which were not examined in this study. A future study could focus on more aspects of aggravating factors that lead to violence against healthcare personnel working in emergency departments. The reduction of these factors along with current measures should also be investigated.

## Conclusion:

Emergency healthcare personnel in metropolitan of Thailand had a high rate of experiencing violence in the previous year, including Thai Emergency physicians whose data had not been explored before. Younger age, less work experience, being a nurse, and working in the urban academic or tertiary emergency department increased the likelihood of being a victim of workplace violence. Only a minority of emergency departments had the recommended violence prevention systems. However, the security measures are not related to workplace violence. 

## Author contribution

NA: Conceptualization, investigation, methodology, major contributor in writing the manuscript, supervision, and funding acquisition. WW: Investigation, data collection, writing – original draft, and visualization. All authors read and approved the final manuscript.

## Competing interests

 The authors declare that they have no competing interests.

## Availability of data and materials:

The data that support the findings of this study are available from the corresponding author, [Adisk Nithimathachoke], upon reasonable request.
